# Effect of interscalene block on intraocular pressure and ocular perfusion pressure

**DOI:** 10.1186/s12871-017-0436-x

**Published:** 2017-10-23

**Authors:** Betul Basaran, Aysun Ankay Yilbas, Zeki Gultekin

**Affiliations:** 1 Department of Anesthesiology, Konya Training and Research Hospital, Meram Yeni Yol street, 42090 Konya, Turkey; 2 Department of Anesthesiology and Reanimation, Hacettepe University, Faculty of Medicine, Ankara, Turkey; 3 Department of Orthopedics and Traumatology, Konya Training and Research Hospital, Konya, Turkey

**Keywords:** Interscalene block, Intraocular pressure, Ocular perfusion pressure

## Abstract

**Background:**

Interscalene block (ISB) is commonly associated with Horner’s syndrome due to spread of local anesthetic to the cervical sympathetic chain. Postganglionic neurons that originate from superior cervical ganglia form the sympathetic innervation of eye. Decrease in sympathetic tone may change intraocular pressure (IOP) and ocular perfusion pressure (OPP). The aim of the study was to investigate whether ISB affects IOP and/or OPP.

**Methods:**

Thirty patients scheduled for ambulatory shoulder surgery under regional anesthesia with a single-shot ISB (15 mL 0.5% bupivacaine and 15 mL 2% lidocaine) were recruited. The IOP and OPP in both eyes, mean arterial pressure (MAP), heart rate (HR) and end-tidal CO_2_ (ETCO_2_) were measured before ISB and 5, 10, 20, 30 and 60 min after ISB in the beach-chair position.

**Results:**

The baseline IOP and OPP were similar in the blocked and unblocked sides (IOP 17.60 ± 1.69 and 17.40 ± 1.96 respectively *p* = 0.432; OPP 49.80 ± 8.20 and 50 ± 8.07 respectively *p* = 0.432). The IOP in the blocked side significantly decreased between 10th to 60th min following ISB, compared to the baseline values (*p* < 0.001). The OPP in the blocked side significantly increased from 10th to 60th min (*p* < 0.001) whereas, there were no significant changes in IOP and OPP throughout the measurement period in the unblocked side.

**Conclusions:**

ISB decreased IOP in the blocked side. ISB could be considered as a safe regional technique of choice in elderly patients at high risk for developing glaucoma.

## Backgrounds

Interscalene brachial plexus block (ISB) commonly produces Horner’s syndrome defined by ipsilateral miosis and ptosis, due to blockade of neighboring cervical sympathetic ganglia [1, 2]. The cervical sympathetic ganglia consist of superior, middle and inferior cervical sympathetic ganglia [3]. The ciliary blood vessels and ciliary epithelium are richly innervated by postganglionic fibers originated from superior sympathetic ganglia [4]. These structures are important determinants of aqueous humor formation. Intraocular pressure (IOP) is regulated by changes in aqueous humor formation and outflow. Sympatholytic effect due to stellate ganglion block (SGB) has been shown to be leading to an acute decrease in IOP of healthy eye and in some types of glaucoma [5–7]. The effect of ISB on IOP is unknown. Importantly, abrupt increase or drop of IOP may have potentially harmful effects especially in elderly patients in whom the risk of undiagnosed glaucoma is high. The aim of this study was to investigate the effect of ISB on IOP in patients receiving ISB for shoulder surgery.

## Methods

This study was performed at Konya Training and Research Hospital, Konya, Turkey. All study documents and procedures were approved by review board at University of Selcuk, Konya, Turkey (protocol no: 2017/ 29; chairperson Dr. Alptekin). Thirty patients with American Society of Anesthesiologists (ASA) physical status I-II, aged 18–65 yr., and scheduled for elective ambulatory shoulder surgery (rotator cuff repair, Bankart repair or subacromial decompression; either arthroscopically or through an open incision) under regional anesthesia with a single-shot ISB were included to the study. Written informed consent was obtained from all patients.

Exclusion criteria were contraindications to ISB (coagulopathies, severe respiratory disease, contralateral phrenic nerve paralysis), hypertension, preexisting acute or chronic eye disease, history of eye surgery, use of drugs known to influence IOP (ß blockers, Ca channel blockers, statins and nitrates), body mass index (BMI) greater than 35 kg/m^2^, preference for general anesthesia or pregnancy, and patient refusal.

In all patients, monitoring consisted of heart rate (HR), noninvasive arterial blood pressure, end-tidal carbon dioxide (ETCO_2_). ISB were performed using combined ultrasound and electrical nerve stimulation technique by the same anesthesiologist experienced with ISB technique (B.B.) (In-line approach with 10-15 MHz probe, Esaote, My Lab 5, Italy). Nerve roots and trunks were visualized in the short axis view at the level of sixth cervical vertebra. After needle position was confirmed with deltoid, biceps, or triceps muscle contraction at threshold current of 0.4-0.6 mA, 30 mL (15 cc %0.5 bupivacaine and 15 cc %2 lidocaine) local anesthetic solution were deposited between fifth and sixth cervical nerve roots by using 22 G 50 mm insulated SonoPlex Stim needle (Pajunk, Germany).

The patients were then assessed every 5 min for 15 min for the development of sensory blockade using ice over the deltoid region by the same anesthesiologist. After 15 min, if loss of sensation to cold was not achieved or incomplete, these patients were excluded from the study.

Horner’s syndrome was defined as ptosis, miosis and scleral and conjunctival injection. Horner’s syndrome onset time was recorded.

IOP measurements were performed with Icare PRO Hand-held tonometer in the beach chair position at all time points. The device shows digital numeric values after each successful contact of the probe with the cornea. When the six measurements in sequence are completed and the measurement valid, the final IOP is displayed. The probe was changed at each patient to avoid contamination. All measurements were performed by the same anesthesia nurse who was trained about hand-held tonometer use.

Ocular perfusion pressure (OPP) was calculated using following equation [8].

$$ \mathrm{OPP}=\left(95/140\times \mathrm{MAP}\right)-\mathrm{IOP} $$

IOP of both eyes, MAP, ETCO_2_ and HR were measured in the beach chair position at baseline (before ISB), 5, 10, 20, 30 and 60 min after ISB, before surgery. ETCO2 were measured during nasal breathing while on room air through nasal sampling cannula.

### Statistical analysis

Data analysis was performed by using IBM SPSS Statistics version 17.0 software (IBM Corporation, Armonk, NY, USA). While, categorical data were shown as number of cases and percentages, descriptive statistics for continuous variables were expressed as mean ± SD or median (min-max), where applicable. The mean differences between the blocked and unblocked sides were compared by paired samples t-test. The repeated measurements of ANOVA by Wilks’ Lambda test were applied for determining whether the differences among measurement times. When the *p*-values from the Wilks’ Lambda test were statistically significant, to know which measurement time differ from which others Bonferroni Adjusted multiple comparison was used. A *p* value less than 0.05 was considered statistically significant. However, all possible multiple comparisons, the Bonferroni Correction was applied for controlling Type I error.

### Sample size estimation

Sample size estimation was performed by using G*Power (Franz Faul, Universität Kiel, Kiel, Germany) version 3.0.10. According to a similar study of Nagahara et al. [5]; the actual difference in ΔIOP at the 10th min according to the baseline, a total sample size of 28 to achieve 85% power to detect a difference of 0.2 mmHg between the null hypothesis that both side means are −0.1 and the alternative hypothesis that the mean of blockage side is 0.1 with estimated standard deviations of 0.2 mmHg for unblocked side and 0.3 mmHg and for blocked side using a two-sided paired samples t-test. The primary aim of this study was to compare the differences in intraocular pressure levels between blocked and unblocked sides, so we decided to recruit a total of 30 patients to allow for drop outs.

## Results

Thirty patients, 15 men and 15 women with mean age of 52.6 ± 10.6 years were included. Twenty patients received right and ten patients received left side ISB (Table 1). All patients showed signs of successful block and ipsilateral Horner’s syndrome. Onset time for Horner’s syndrome was 3 (1–8) min. There were no differences in MAP, HR and ETCO_2_ as compared to the baseline values (Table 2).

**Table 1:** Demographical and clinical characteristics

	*n* = 30
Age (years)	52.6 ± 10.6
Range of age (years)	29-65
Gender	
Female	15 (50.0%)
Male	15 (50.0%)
Weight (kg)	72.7 ± 11.1
Height (cm)	166.3 ± 8.4
Body mass index (kg/m^2^)	26.2 ± 2.8
Block side	
Right	20 (66.7%)
Left	10 (33.3%)
Onset time of Horner syndrome (min)	3 (1-8)

Values are presented as mean ± SD, range or absolute number (%), as appropriate

**Table 2:** Hemodynamic measurements regarding for follow-up times

	HR	MAP	ETCO_2_
Baseline	81.70 ± 11.92	99.33 ± 13.15	35.47 ± 1.43
5th min	79.93 ± 11.83	96.70 ± 10.88	35.63 ± 1.10
10th min	80.77 ± 12.46	94.77 ± 11.89	35.80 ± 0.85
20th min	79.70 ± 10.63	96.53 ± 10.86	35.83 ± 1.29
30th min	78.90 ± 9.75	94.80 ± 9.35	35.87 ± 0.73
60thmin	77.33 ± 7.93	94.40 ± 8.43	35.87 ± 0.94
p-value^†^	0.220	0.140	0.807

Values are presented as mean ± SD. *HR* heart rate (/minute), *MAP* mean arterial pressure (mmHg), *ETCO*_*2*_ End-tidal CO_2_(mmHg)

^†^Comparisons among measurement times, Repeated Measurement of Variance analysis by Wilks’ Lambda test, *p* < 0.05 was considered as statistically significant

**Fig. 1:**
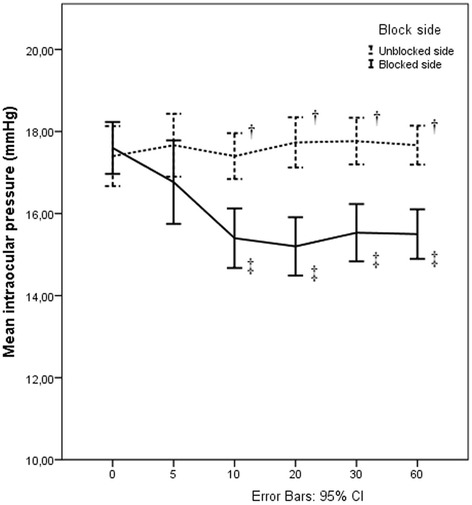
Changes in intraocular pressure in the blocked and unblocked sides. Measurements were made before interscalene block (ISB) (0), 10, 20, 30, 60 min after ISB (10), (20), (30), (60) respectively. Vertical bars denote 0.95 confidence intervals. † Statistically significant comparisons between the blocked and unblocked sides within each measurement time 10th min *p* < 0.001, 20th min *p* < 0.001, 30th min *p* < 0.001, 60th min *p* < 0.001 (Paired Samples t-test, *p* < 0.05 was considered as statistically significant) ‡ Statistically significant comparisons with baseline within the blocked side, (Repeated Measurement of Variance analysis by Wilks’ Lambda test, *p* < 0.05 was considered as statistically significant)10th min (*p* < 0.001), 20th min (*p* < 0.001), 30th min (*p* < 0.001), 60th min (*p* < 0.001)

Baseline IOP in the blocked and unblocked sides were similar (17.60 ± 1.69 and 17.40 ± 1.96 mmHg for blocked and unblocked sides respectively, *p* = 0.432). The IOP in the blocked side was significantly reduced between 10th and 60th min following ISB compared to the baseline value (*p* < 0.001) while the IOP in the unblocked side remained unchanged (Fig. 1). The difference between the blocked and unblocked sides in the time course of the ΔIOP was significant at all time points (*p* < 0.001) (Table 3).

**Table 3:** Actual differences in intraocular pressures in comparison with baseline values

	Blocked side	Unblock side	p-value^†^
ΔIOP 5 min	−0.83 ± 2.05	0.27 ± 1.28	*< 0.001*
ΔIOP 10 min	−2.20 ± 1.56	0.00 ± 1.17	*< 0.001*
ΔIOP 20 min	−2.40 ± 1.48	0.33 ± 1.49	*< 0.001*
ΔIOP 30 min	−2.07 ± 1.60	0.37 ± 1.32	*< 0.001*
ΔIOP 60 min	−2.10 ± 1.47	0.27 ± 1.31	*< 0.001*

Values are presented as mean ± SD. *IOP* intraocular pressure (mmHg). ΔIOP: indicates difference in IOP between before and each time point of measurement

^†^Paired Samples t-test, *p* < 0.05 was considered as statistically significant results (italic)

**Fig. 2:**
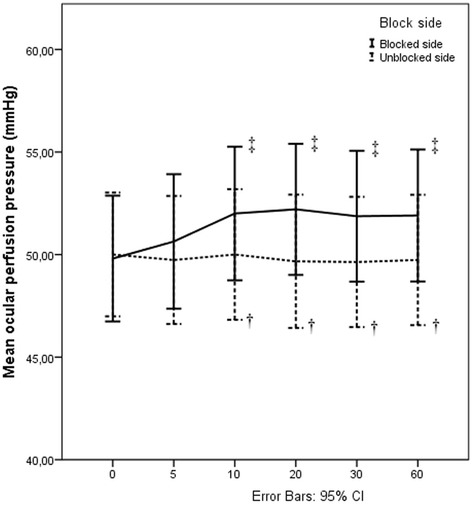
Changes in ocular perfusion pressure in the blocked and unblocked sides. Measurements were made before interscalene block (ISB) (0), 10, 20, 30, 60 min after ISB (10), (20), (30), (60) respectively. Vertical bars denote 0.95 confidence intervals. † Statistically significant comparisons between the blocked and unblocked sides within each measurement time 10th min *p* < 0.001, 20th min *p* < 0.001, 30th min *p* < 0.001, 60th min p < 0.001 (Paired Samples t-test p < 0.05 was considered as statistically significant), ‡ Statistically significant comparisons with baseline within the blocked side, (Repeated Measurement of Variance analysis by Wilks’ Lambda test, p < 0.05 was considered as statistically significant)10th min (p < 0.001), 20th min (p < 0.001), 30th min (p < 0.001), 60th min (p < 0.001)

The OPP in the blocked side was significantly increased between10th and 60th min following ISB compared to the baseline value (*p* < 0.001). On the other hand, the OPP in the unblocked side showed no significant difference at all time points compared to the baseline. In addition, OPP in the blocked side was significantly higher from unblocked side between 10th and 60th min following ISB (*p* < 0.001) (Fig. 2).

## Discussion

In the present study, we found that ISB produced significant decreases in IOP of blocked side eye in patients with beach chair position. Moreover, OPP was preserved in the unblocked side while OPP in the blocked side increased following ISB.

The use of ultrasound leads to decrease the need for local anesthetic dose and related complications. However, the success rate of ISB with low dose local anesthetics in everyday clinical practice may be inferior to those reported in meticulously conducted clinical trials; 20-30 ml volume of local anesthetic dose is still used and related complications including Horner’s syndrome can be seen [9].

**Fig. 3:**
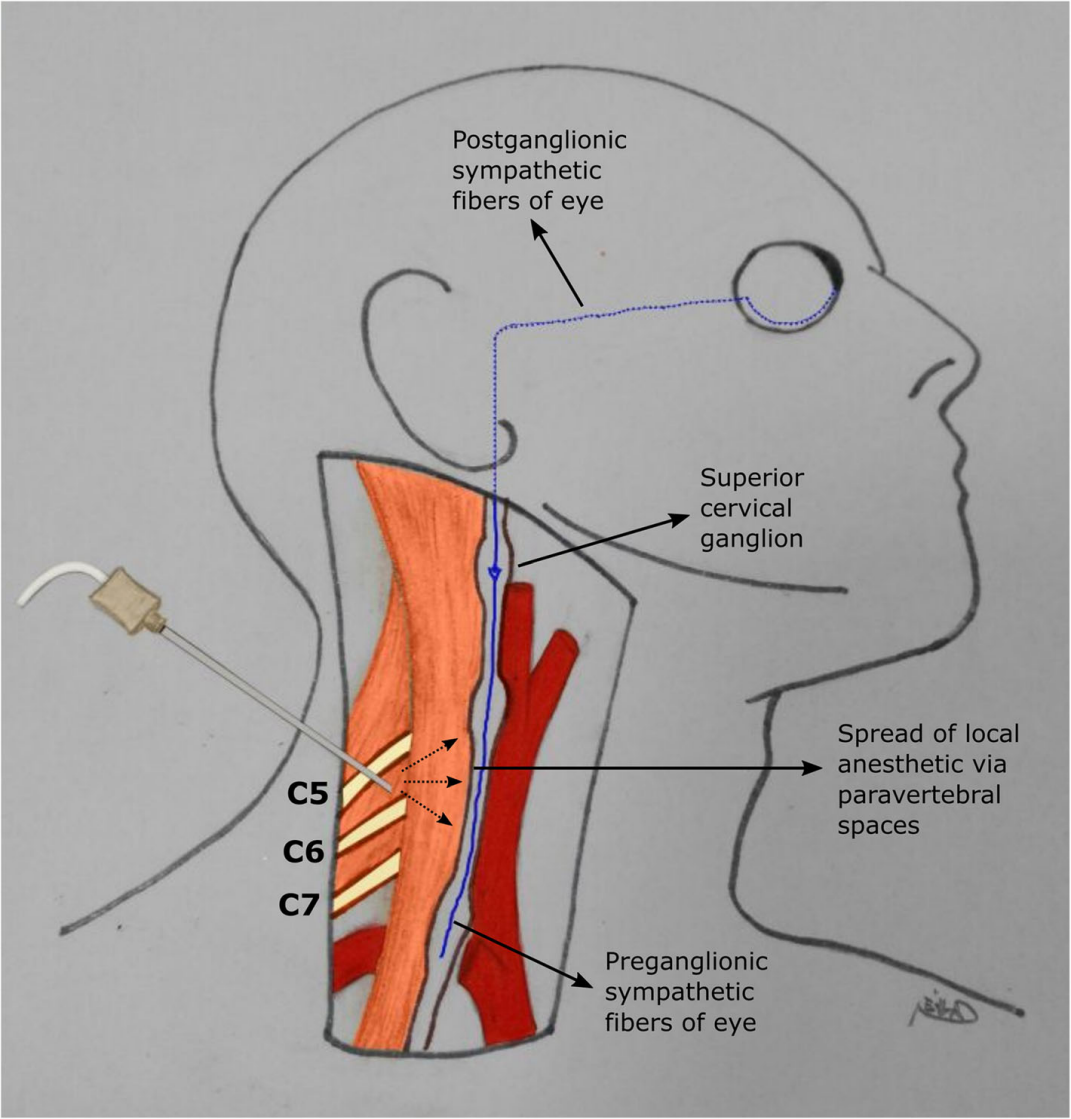
The described pathway of local anesthetic spread

Cervical sympathetic trunk is embedded in the deep fascia between carotid sheath and the prevertebral layer of deep fascia. It is located close to the spinal roots of the brachial plexus. During ISB, diffusion of large volume of local anesthetic in prevertebral spaces results in paralysis of neighboring cervical sympathetic chain via communication between interfascial spaces (Fig. 3). The presence of Horner’s syndrome is an indication for the spread of local anesthetics to the cervical sympathetic ganglia. The ocular projections of superior cervical ganglia, part of cervical sympathetic ganglia, influence many functions of the eye including ocular blood flow and IOP. There are a number of studies indicating that stellate ganglion block (SGB) is associated with increased ipsilateral eye retinal circulation with or without IOP change [5, 10]. In the present study, while ipsilateral IOP decreased significantly 10 min after ISB, the contralateral eye IOP was unchanged. These results are consistent with results of previous studies which aimed to search the effect of SGB on retinal circulation and IOP [6]. SGB and ISB would both affect the ascending preganglionic neurons of superior sympathetic ganglion or the ganglion itself due to its near location to the blocked sides.

ISB can be utilized as a sole anesthetic technique or in combination with general anesthesia for shoulder surgery. Regional anesthesia has been advocated for several advantages over general anesthesia. These include shorter hospital stay, reduced postoperative analgesia requirement and avoidance of risks and side effects of general anesthesia [11–13]. It could be the anesthetic choice especially in elderly population with underlying comorbidities. Vascular diseases such as atherosclerosis, vasospastic diseases and diabetes causes dysfunctional auto-regulation of ocular blood flow, likely contributing to glaucomatous optic neuropathy [14]. It is well known that, even transient episodes of increased IOP may jeopardize retinal perfusion and cause retinal ischemia in older patients with atherosclerotic involvement of retinal artery [15]. The incidence of glaucoma is also known to increase six times with age. Therefore, any intervention that is intended for use in elderly patients should not increase intraocular tension [16]. On the downside, sudden deep decrease of IOP can actually lead to bleeding in patients with very high intraocular pressure [17]. We have shown that ISB causes a slight decrease in IOP of blocked side and does not change in unblocked side eye. However, further studies are required in patients with preexisting eye disorders. Similarly; IOP may decrease in primary open angle glaucoma, early secondary glaucoma and postoperative ocular hypertension following SGB, whilst the IOP of eyes with primary angle closure glaucoma and advanced secondary glaucoma appear to remain elevated [18]. In addition, repeated stellate ganglion blocks have been reported to aggravate glaucoma, thus, they are considered as a relative contraindication [19]. In this context, further studies are needed to delineate the effects of ISB in patients with certain types of glaucoma.

Beach chair position combined with or without induced hypotension results in reduction of cerebral perfusion pressure; therefore, ocular perfusion pressure would drop in this position. [20–23]. It is possible that cerebral perfusion pressure may be better maintained under regional anesthesia [24]. By this way OPP also would be preserved. Recent population based studies have described the potential effects of low ocular perfusion pressure in the development and progression of glaucoma [25, 26]. For this reason, OPP maintenance during perioperative period has gained importance among patients with low OPP values. According to our results, a significant decrease in OPP does not seem to be expected after ISB in both blocked and unblocked eyes. Significantly decreased IOP and modestly decreased NIBP translated into a significant difference in OPP between the blocked and unblocked sides. This showed that ISB maintains OPP despite the variations in the MAP.

This study has some limitations. Firstly, we did not objectively assess blockade of superior sympathetic ganglion by spread of local anesthetic with any acceptable method. Accordingly, we can’t estimate in what extend the fibers which are responsible for sympathetic innervations of the eye were affected. Secondly, we aimed at showing the IOP changes after ISB in patients without systemic hypertension. But high systemic blood pressure is associated with increased IOP [27]. Thirdly, our study aimed at showing the acute effect of ISB within the first 1 hour after the block performance. Therefore, it is difficult to precisely define later effects of ISB on IOP and OPP. There may be rebound rise in IOP of affected side. Lastly, the results of the current study are only valid for relatively healthy patients. Hence, the findings of this study can’t be directly extrapolated to glaucoma patients.

## Conclusions

In conclusion, we have demonstrated that interscalene brachial plexus block performed with 30 mL local anesthetic solution was associated with a drop in IOP in the blocked side, while IOP in the unblocked side remained unchanged. This study might represent a new step in the use of ISB as it shows that decrease in IOP. Although further studies including elderly patients with glaucoma are needed, our results suggest that ISB might have another advantage in patients with high intraocular pressure.

## Abbreviations

ASA: American Society of Anesthesiologists physical status
ETCO_2_: End-tidal CO_2_
HR: Heart rate
IOP: Intraocular pressure
ISB: Interscalene block
MAP: Mean arterial pressure
min: Minute
OPP: Ocular perfusion pressure
SGB: Stellate ganglion block
